# Randomised controlled trial of local corticosteroid injections for de Quervain's tenosynovitis in general practice

**DOI:** 10.1186/1471-2474-10-131

**Published:** 2009-10-27

**Authors:** Cyriac Peters-Veluthamaningal, Jan C Winters, Klaas H Groenier, Betty Meyboom-deJong

**Affiliations:** 1 Department of General Practice, University Medical Center Groningen, Groningen, the Netherlands

## Abstract

**Background:**

De Quervain's tenosynovitis is a stenosing tenosynovitis of the first dorsal compartment of the wrist and leads to wrist pain and to impaired function of the wrist and hand. It can be treated by splinting, local corticosteroid injection and operation. In this study effectiveness of local corticosteroid injections for de Quervain's tenosynovitis provided by general practitioners was assessed.

**Methods:**

Participants with de Quervain's tenosynovitis were recruited by general practitioners. Short-term outcomes (one week after injections) were assessed in a randomised, placebo-controlled trial. Long-term effectiveness was evaluated in an open prospective cohort-study of steroid responders during a follow-up period of 12 months. Participants were randomised to one or two local injections of 1 ml of triamcinolonacetonide (TCA) or 1 ml of NaCl 0.9% (placebo). Non-responders to NaCl were treated with additional TCA injections. Main outcomes were immediate treatment response, severity of pain, improvement as perceived by participant and functional disability using sub items hand and finger function of the Dutch Arthritis Impact Measurement Scale (Dutch AIMS-2-HFF).

**Results:**

11 general practitioners included 21 wrists in 21 patients. The TCA-group had better results for short-term outcomes treatment response (78% vs. 25%; p = 0.015), perceived improvement (78% vs. 33%; p = 0.047) and severity of pain (4.27 vs. 1.33; p = 0.031) but not for the Dutch-AIMS-HFF (2.71 vs. 1.92; p = 0.112). Absolute risk reduction for the main outcome short-term treatment response was 0.55 (95% CI: 0.34, 0.76) with a number needed to treat of 2 (95% CI: 1, 3). In the cohort of steroid responders (n = 12) the beneficial effects of steroid injections were sustained during the follow-up of 12 months regarding severity of pain (p = 0.67) and scores of Dutch AIMS-2-HFF (p = 0.36), but not for patient perceived improvement (p = 0.02). No adverse events were observed during the 12 months of follow-up.

**Conclusion:**

One or two local injections of 1 ml triamcinolonacetonide 10 mg/ml provided by general practitioners leads to improvement in the short term in participants with de Quervain's tenosynovitis when compared to placebo. The short-term beneficial effects of steroid injections for symptoms were maintained during the follow-up after 12 months.

**Trial registration:**

Current Controlled Trials ISRCTN53171398

## Background

De Quervain's tenosynovitis is a condition that causes wrist pain and that can lead to disfunction of the affected hand. It is caused by impaired gliding of the tendons of the abductor pollicis longus (APL) and extensor pollicis brevis (EPB) muscles[1]. This is most probably caused by thickening of the extensor retinaculum (the thickened part of the general tendon sheath that holds the tendons of the extensor muscles in place) of the wrist.

Fritz de Quervain, a Swiss physician, is given credit for first describing this condition with a report of five cases in 1895 and eight additional cases in 1912[2,3]. Although the term stenosing tenosynovitis is frequently used, pathophysiology of de Quervain's disease does not involve inflammation since on histopathological examination mainly degenerative changes such as myxoid degeneration, fibrocartilagenous metaplasia and deposition of mucopolysaccharide are seen[4].

The diagnosis is made by history and physical examination. Symptoms consist of pain or tenderness at the radial styloid sometimes radiating to the thumb, forearm or shoulder and on physical examination there might be swelling at the radial styloid with tenderness and crepitations on palpation. Finkelstein's test (deviating the wrist to the ulnar side, while grasping the thumb, results in pain) is in typical cases positive. A positive Finkelstein's test has a between observer repeatability (k) of 0.79[5]. Unfortunately there is no golden diagnostic confirmatory test for de Quervain's tenosynovitis. In the literature a variety of terminology (e.g. tendinitis, peritendinitis, tenosynovitis, tenovaginitis) and case-definitions are used for this condition. In 1998 and 2001 efforts have been made to construct reliable classifications and case-definitions for soft-tissue rheumatic disorders of the upper limb, including de Quervain's tenosynovitis [5-7].

In a large community based study from the United Kingdom, the prevalence of de Quervain's tenosynovitis was 0,5% for men and 1,3% for women[8]. It was associated with considerable impact on daily activities and health seeking behaviour. The prevalence and incidence of patients with de Quervain's tenosynovitis in primary care are not known.

De Quervain's tenosynovitis can be treated by operative and non-operative treatment. Operative therapy (slitting or removing a strip of the tendon sheet) has been reported to be effective with a 91% cure rate in non-controlled studies, but is more invasive and associated with higher costs and the possibility of surgical complications[9].

The effectiveness of injection therapy is often attributed to anti-inflammatory effects of corticosteroids but the exact mechanism of action remains unclear. A recent Cochrane review found one controlled clinical trial by Avci of 18 participants (all pregnant or lactating women) that compared one steroid injection with methylprednisolone and bupivacaine to splinting with a thumb spica[10,11]. All patients in the steroid injection group (9/9) achieved complete relief of pain whereas none of the patients in the thumb spica group (0/9) had complete relief of pain, one to six days after intervention (number needed to treat to benefit (NNTB) = 1. However, it was also concluded that the applicability of these findings to daily clinical practice may be limited, as they were based on only one trial with a small number of included participants, the methodological quality was poor and only pregnant and lactating women participated in the included study. In another systematic review of effectiveness of corticosteroid injection for de Quervain's tenosynovitis that included seven observational studies (with totally 459 wrists), 83% of the 226 wrists that received injection alone were cured, 61% of the 101 wrists that received injection and splint immobilization were cured and only 14% of those who received splinting alone were cured[12]. A study that compared effectiveness of one injection of 10 mg triamcinolonacetonide injection to triamcinolonacetonide injections combined with 200 mg oral nimesulide during seven days found no significant differences between the two groups and success rate after three weeks was 67% in the nimesulide + triamcinolone group and 68% in the triamcinolone alone group[13].

Other conservative treatment modalities, such as heat, cold, diathermy, strapping, splints, rest, massage, counterirritants and medications were found not to be effective[1].

The lack of high quality controlled trials and data regarding effectiveness of local corticosteroid injections provided by general practitioners prompted us to design a randomised, placebo controlled trial in primary care to evaluate effectiveness of local steroid injections for de Quervain's tenosynovitis.

## Methods

This trial is part of a larger study called the Groningen Hand and Wrist Injection Therapy Trial (HAWITT) in which efficacy, safety and feasibility of corticosteroid injections for carpal tunnel syndrome, de Quervain's tenosynovitis and trigger finger in general practice were assessed. In this report the results for de Quervain's tenosynovitis are described.

Approval for the HAWITT-study was given by the medical ethics committee of the University Medical Center Groningen (METc 2002/020c).

### Setting

Patients were recruited from the practices of 11 general practitioners in the northern part of the Netherlands.

### Patient recruitment, in- and exclusion criteria

Patients presenting to the participating general practitioners with a clinical diagnosis of de Quervain's tenosynovitis were recruited in the period of 2003-2005. A clinical diagnosis of de Quervain's tenosynovitis was defined as pain or tenderness at the radial styloid combined with either a positive Finkelsteins test or crepitations on palpation at the radial styloid.

Exclusion criteria were being of minor age (< 18), presence of an absolute contraindication for corticosteroid injection, prior treatment in the last six months with steroid injection and/or surgery at the same anatomical location, possible traumatic or neoplastic origin of symptoms, inability to fill in follow-up forms or absence of self determination in the participating patient. After written informed consent was obtained by the patient's general practitioner, baseline data were collected and patients were randomised to either steroid or saline injection.

### Intervention and injection technique

Participants received one or two local injections with either 1 ml triamcinolonacetonide 10 mg/ml (experimental intervention) or 1 ml NaCl 0.9% (control intervention). One millilitre of the trial medication was deposed after inserting the needle along the line of the tendon, just proximal or distal to the styloid, at the site of maximum tenderness.

All general practitioners involved in the study were offered a two-hour course on diagnosing de Quervain's tenosynovitis and the technique of injection therapy, using an arm phantom for instruction.

### Randomisation and allocation concealment

Randomisation was realized using an electronic online randomisation tool developed by G. Urbaniak (, accessed on 22.12.2002). Block randomisation was performed by creating 5 sets of blocks of 10 random numbers. Even numbers corresponded with active trial medication and uneven numbers with placebo to ensure equal numbers of allocation to active and placebo treatment. Treatment allocation was written on a paper and enclosed in an opaque and sealed envelope. After inclusion of a patient a pharmacy assistant at a remote location who was not involved in the study drew an envelope and sent the allocated trial medication to the injecting general practitioner.

### Study design, blinding and bail-out treatment

Every patient with a clinical diagnosis of de Quervain's tenosynovitis presenting to one of the participating general practitioners was asked to participate in the trial. After applying inclusion and exclusion criteria assessment of baseline clinical characteristics took place by the patient's own general practitioner, who also performed the blinded assessment of the short-term follow-up two weeks after the intervention. In order to guarantee blinding of short-term outcome assessment (after randomisation) the trial medication was injected one week after inclusion by a second independent general practitioner. If the result of the first injection was not satisfactory in the participant's opinion, the participants were given a second injection by the other independent general practitioner one week later. Two weeks after the injections with the trial medication the participants were instructed to return to their own general practitioner for assessment of short-term outcomes. Because a placebo look-alike of the triamcinolonacetonide injection suspension could not be manufactured, blinding was realised by applying the injection while the participant was blindfolded.

### Bail-out treatment

If during short term outcome assessment the response to the blinded injection(s) was insufficient according to agreement between the patient and general practitioner blinding was discontinued and the trial centre was asked whether injected trial medication consisted the active substance (TCA) or control treatment (NaCl). Participants who were randomized to TCA with no response to blinded injections were referred to secondary care for operative treatment and not included in the long-term analysis.

In case of insufficient response after injection of NaCl, one or two additional injections with TCA (bail-out treatment) with weekly intervals were given without blinding. In case of insufficient response to one or two bailout injections, participants were referred to secondary care for operative treatment and not included in the long-term follow-up analysis. Introducing bailout treatment for non-responders to NaCl was required, as the medical ethics committee considered it to be unethical to leave patients, who received placebo treatment with no improvement in symptoms after intervention, untreated.

### Outcomes measurements

During short term assessment primary outcome measurements were recorded. Primary outcomes were:

1. direct treatment response (based on consensus between physician and participant), one week after injection treatment:

• 0 = no response

• 1 = partial response, but not satisfactory, warranting further treatment

• 2 = partial response, satisfactory, not warranting further treatment

• 3 = complete resolution of symptoms and signs

2. severity of pain at the radial styloid during the week prior to measurement using a numerical rating scale: 0 = no pain; 10 = severe pain

3. Functional status was recorded by using the sub items hand and finger function of the Dutch version of the second version of the Arthritis Impact Measurement Scale (DUTCH AIMS-2-HFF)[14].

4. improvement as perceived by participant, using a 5-point ordinal scale:

• -2 = much worse

• -1 = worse

• 0 = not better/not worse

• +1 = better

• +2 = much better

The occurrence of adverse events (quantitative and qualitative) at short-term assessment was a secondary outcome.

Follow up measurements were performed by sending questionnaires to participating patients 1, 3, 6 and 12 months after the last injection and consisted of the same primary outcome measures as during short term assessment except for direct treatment response.

### Sample size and data analysis

Calculations of sample size were based on a two-sided alpha of 0.05, a statistical power of 0.90. The proportion of patients treated with steroid injection with satisfactory response or complete resolution after two injections was expected to be at least 70%, extrapolated from prior prospective studies. Adequate treatment response to placebo treatment was expected to be 20%. Based on these calculations we aimed to recruit 25 patients for each treatment group. An intention tot treat analysis was used.

For continuous data the student T-test was used if the distribution was normal and Mann-Witney U test if there was not a normal distribution. For categorical data Fisher's exact test was used. Friedmann's test was used to compare observations repeated on the same subjects and to test if the distributions are the same across repeated measures if a non-normal distribution of outcome data was suspected. Significance was accepted at a probability value of < 0.05. To calculate the number to treat the formula NNT = 1/ARR was used, where ARR (Absolute Risk Reduction) = CER (Control Group Event Rate) - EER (Experimental Group Event Rate). Missing values were imputated using the expectation maximation score. Data were analysed using the statistical software SPSS version 14 (SPSS Inc Chicago, Illinois, USA).

## Results

### Inclusion of participants and baseline characteristics

**Figure 1:**
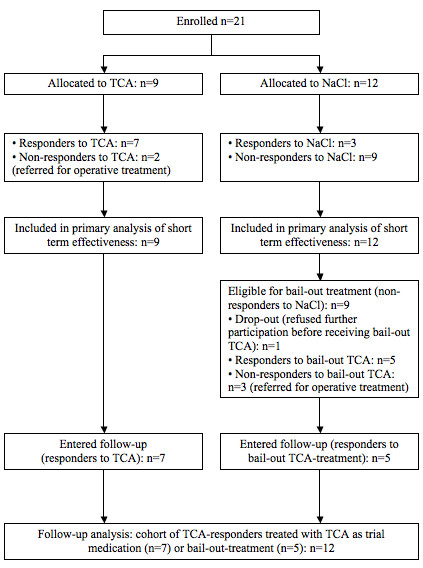
**flowchart: flow of participants**. TCA = 1 ml triamcinolonacetonide 10 mg/ml NaCl = 1 ml NaCl (0.9%).

During the inclusion period of 2 years (January 2003 to January 2005) 21 participants who fulfilled inclusion criteria were recruited by 11 general practitioners of the HAWITT-trial. At baseline assessment the two intervention-groups were found to be comparable regarding potentially prognostic indicators and differed only in male to female ratio (table 1). After randomisation 9 patients were allocated to TCA and 12 to NaCl (figure 1).

**Table 1:** Baseline characteristics of participants with de Quervain's tenosynovitis

	**NaCl (n = 12)**	**TCA(n = 9)**
mean age (SD)	52.3 (12.6)	51.2 (20.2)
sex (female/male)*	10/12	3/9
median (min, max) duration of symptoms (weeks)	8 (2, 50)	5 (3, 24)
affected side (right/left)	8/3	4/5
dexterity (right/left)	11/0	7/2
Median (min, max) Dutch AIMS-HFF score	2.6 (1.0, 3.6)	2.4 (1.0, 4.5)
Median (min, max) severity of pain	7 (4, 10)	7 (2, 8)

* p value = 0.32 (Fisher's Exact test)

SD = standard deviation

NaCl = NaCl 0.9% (saline)

TCA = triamcinolonacetonide 10 mg/ml

### Short-term efficacy

**Figure 2:**
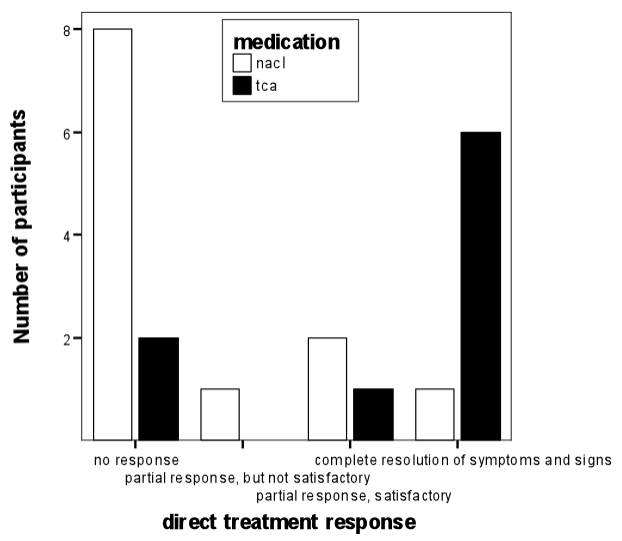
**Immediate treatment response one week after one or two injections**.

In table 2 and figure 2 the results of outcomes one week after the last injection are displayed. The TCA-group showed better outcomes than NaCl-group for the direct treatment response (p = 0.02), severity of local pain (1.3 {95% CI: 0.3, 2.9} vs 4.3 {95% CI: 2.3, 6.3}; p = 0.03) and perceived improvement (p = 0.047) but not for total scores of Dutch AIMS-2 sub items hand- and finger function (1.9 {95% CI: 0.7, 3.2} vs. 2.7 {95% CI: 1.9, 3.6}; p = 0.11). Using direct treatment response as the main outcome measure absolute risk reduction was 0.55 (95% CI: 0.34, 0.76) with a number needed to treat of 2 (95% CI: 1, 3).

**Table 2:** Short term results of 1-2 local injections for de Quervain's tenosynovitis (1 week after last injection)

		**NaCl (n = 12)**	**TCA (n = 9)**	**p**
direct treatment response	no response	8	2	
	partial response, not satisfactory	1	0	
	partial response, satisfactory	2	1	
	complete resolution of symptoms	1	6	
				0.015*

mean severity of pain in the past week (95% CI)		4.3 (2.3-6.3)	1.3 (0.3-2.9)	
	mean rank	13.1	7.4	0.031^§^

mean Dutch AIMS-2-HFF (95% CI)		2.7 (1.9-3.6)	1.9 (0.7-3.2)	
	mean rank	12.4	8.2	0.112^§^

patient perceived improvement	much worse	0	0	
	worse	2	0	
	not better not worse	6	2	
	better	1	1	
	much better	3	6	
				0.047*

*Chi-Square test for linear trend

^§^Mann-Whitney U test

NaCl = NaCl 0.9% (saline)

TCA = triamcinolonacetonide 10 mg/ml

### Long-term efficacy

**Figure 3:**
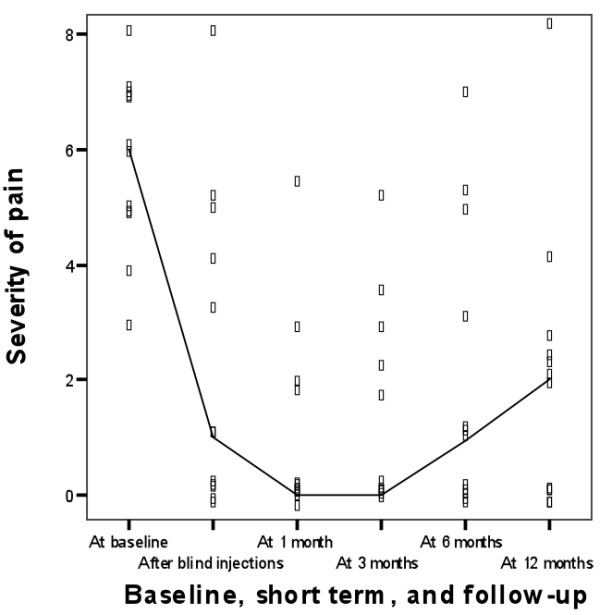
**Severity of pain during follow-up of 12 months**. Lines connect medians.

**Figure 4:**
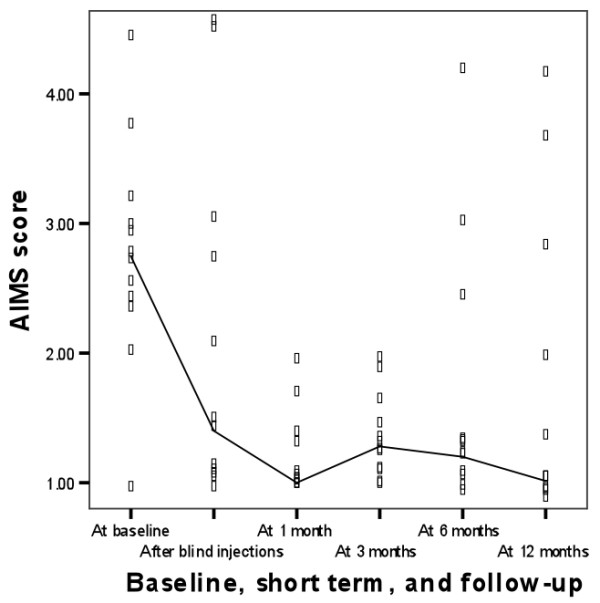
**Dutch AIMS-2-HFF score during follow-up of 12 months**. Lines connect medians.

In table 3 and figures 3 and 4 the results for long-term outcomes are displayed. Since non-responders to NaCl required subsequent bail-out injections with TCA and non-responders to TCA-injections (blinded or bail-out) were referred to secondary care for operative treatment, we present the long term results as a cohort study of patients that had responded to treatment with TCA.

**Table 3:** Long-term results of TCA-responders (n = 12) after 1-2 TCA-injections during follow-up of 12 months

**Follow-up period**									
		
	**1 month**		**3 months**		**6 months**		**12 months**		p-value*
Original study arm	TCA (N = 7)	NaCl (N = 5)	TCA (N = 7)	NaCl (N = 5)	TCA (N = 7)	NaCl (N = 5)	TCA (N = 7)	NaCl (N = 5)	

mean severity of pain (SD)	1.33 (2.05)	1.20 (1.65)	1.25 (1.88)	1.98 (1.82)	2.55 (2.98)	1.32 (1.41)	2.47 (2.93)	1.41 (1.31)	0.67

mean score Dutch AIMS-2-HFF (SD)	1.20 (0.25)	1.30 (0.45)	1.24 (0.29)	1.47 (0.34)	1.59 (1.16)	1.82 (0.85)	1.50 (0.99)	2.44 (1.27)	0.36

mean perceived improvement (SD)	1.44 (0.95)	1.38 (0.90)	1.84 (0.37)	1.25 (0.73)	1.43 (0.73)	1.01 (1.04)	1.60 (0.73)	1.03 (1.01)	0.02

*Friedman test

SD = standard deviation

TCA = triamcinolonacetonide 10 mg/ml

In the NaCl-group three non-responders to NaCl dropped out because they declined further participation in the study. They did not receive bailout TCA-injections and could not be included in the long-term analysis.

The treatment effects of local injections were upheld in the cohort of participants that responded to TCA injections (n = 12) during the follow-up period of twelve months for the outcomes severity of pain (p = 0.67) and Dutch AIMS-HFF score (p = 0.36), but not for patient perceived improvement (p = 0.02).

### Side-effects and serious adverse events

No serious adverse events such as tendon ruptures or deep skin infections were observed. The most frequent reported minor side effects were hot flushes (n = 2) and steroid-flare (n = 6).

## Discussion

Our study indicates that one or two local injections with 1 ml of triamcinolonacetonide 10 mg/ml provided by general practioners leads to improvement of symptoms one week after injection when compared to placebo injections. The effect size was considerable with a number to treat of two.

The effects of steroid injections on functional status of the hand and wrist were less impressive in this study. Also the evidence emerging from this study for long term effectiveness up to one year is less strong than for short term effectiveness, since we were only able to present long term data for the cohort of steroid responders and short term beneficial effects were only maintained during follow-up for the severity of pain and the score for the Dutch AIMS-2 sub items hand and finger function and not for patient perceived improvement. We observed only a few minor side effects.

Unfortunately we were not able to recruit as many participants as we planned. According to the sample-size calculation, inclusion of 50 participants was required, while we were only able to include 21 patients. Difficulties with recruitment of patients for scientific studies in general practice, especially when incident cases are required, is an acknowledged problem and this phenomenon is known as Lasagna's law[15]. Since data on incidence of de Quervain's tenosynovitis in primary care in the Netherlands were not available, it was difficult for us to predict at the start of the study whether we would be able to recruit enough participants.

At baseline assessment the TCA-group and the NaCl-group were found to be comparable regarding the most important potentially prognostic indicators and differed only in male:male ratio, with males overrepresenting the TCA-group.

If we compare our study to the only other controlled study (Avci) available so far that assessed effectiveness of corticosteroid injections for de Quervain's tenosynovitis the following strong points can be noted in our study: inclusion was not restricted to pregnant or lactating women, we used rigorous procedures for allocation concealment and randomisation, participants and outcome assessors were blinded, multiple and clearly defined outcome-measures were used, long term sustainability of effectiveness was also assessed and our study is the first study using a primary care study population.

The finding of a success-rate of 78% of steroid injections is consistent with the success rates found by Jirarattanaphochai (67-68%) and the systemic review of non-controlled studies by Richie (83%), but lower than in the controlled trial by Avci (100%), which was the only study that was included in a Cochrane review [10,12]. A possible explanation for the difference between the results of the study by Avci and our study could be that the risk of bias is probably smaller in our study, since we used more rigorous procedures for allocation concealment and randomisation and participants and outcome assessors were blinded. Bias due to inadequate allocation concealment and randomisation is known to result in overestimation of treatment effects[16].

We found poorer outcomes for functional status at short-term assessment using the Dutch AIMS2-HFF measurement tool, which contrasted in relation to the better outcomes for treatment response, severity of pain and patient perceived improvement. This could be explained by the recent finding by Spies-Dorgelo et al that the AIMS-2-HFF had poor measurement properties regarding reliability and responsiveness in a cohort of participants with hand and wrist complaints in general practice[17]. Maybe the AIMS-2-HFF is not an adequate outcome-tool to assess functional limitations caused by hand and wrist disorders in primary care and to evaluate treatment effects.

Our study indicates that local injection therapy with corticosteroids for de Quervain's tenosynovitis provided by a primary care provider is an effective alternative to surgical therapy and we suggest that initial treatment should be injection therapy with 1-2 local injections with corticosteroids and in case of insufficient response or recurrence the patient should be referred for surgical treatment.

## Conclusion

One or two local injections of 1 ml triamcinolonacetonide 10 mg/ml is an effective method of treatment provided by general practitioners for de Quervain's tenosynovitis with respect to short term outcomes when compared to placebo injection. The short-term effects were maintained for most of the outcome measures during the follow-up period of 12 months, but this was based on outcomes of the cohort of steroid responders and thus long term effectiveness is less clear.

## Competing interests

The authors declare that they have no competing interests.

## Authors' contributions

CPV is the guarantor and was responsible for daily project-management, trial-design, trial-logistics, data-collection and text of the paper and was supervised during these processes by JCW and BMJ. JCW initiated the study, obtained funding, contributed to the trial design and text of the paper. KHG perfomed the statistical analyses and BMJ contributed to the design of the trial and text of the paper. All authors read and approved the final manuscript.

## Pre-publication history

The pre-publication history for this paper can be accessed here:


